# Diagnostic Inaccuracies of Barrett’s Oesophagus on Gastroscopy: Are We Performing Unnecessary Surveillance?

**DOI:** 10.7759/cureus.9850

**Published:** 2020-08-18

**Authors:** Conor Brosnan, Enda Hannan, William Duggan, Tim Harding, Donal Maguire, Anthony T Stafford

**Affiliations:** 1 General Surgery, St. Michael's Hospital, Dublin, IRL; 2 Colorectal Surgery, Royal College of Surgeons in Ireland, Dublin, IRL

**Keywords:** barrett's oesophagus, barrett’s dysplasia, gastroscopy, surveillance, upper endoscopy, oesophageal cancer, git endoscopy, surgical endoscopy

## Abstract

Background

It is common for patients to enter Barrett’s oesophagus (BO) surveillance based on endoscopic appearances before the diagnosis is histologically confirmed. We set out to review this practice by establishing the accuracy of endoscopic diagnoses of BO.

Methods

All gastroscopy reports in which a diagnosis of BO was recorded were reviewed over one year. These were compared to the histopathological reports to assess diagnostic accuracy.

Results

BO was diagnosed in 84 procedures. This diagnosis was incorrect according to histology in 42.9% (n=36) of cases. Diagnostic accuracy was higher with gastroenterologists (38.8% incorrect, n=21) compared to surgeons (50% incorrect, n=15). Diagnostic accuracy was higher with consultants (34.9% incorrect, n=22) compared to registrars (66.7% incorrect, n=14). The dose of sedation used had no impact on accuracy. Unnecessary surveillance was booked in 36.1% (n=13) of cases.

Conclusion

It is insufficient to rely on endoscopic appearances alone to diagnose BO, irrespective of speciality or experience. The diagnosis should only be made after reviewing the histopathology report. This can eliminate unnecessary repeat endoscopy procedures, sparing patients from unjustifiable risk and helping to cut down on long waiting lists in endoscopy departments. The implementation of the Prague classification and Seattle protocol can improve diagnostic accuracy.

## Introduction

Barrett’s oesophagus (BO) refers to intestinal metaplasia of the lower oesophagus, with replacement of normal stratified squamous epithelium by columnar epithelium [1]. It is a widely recognised complication of gastroesophageal reflux disease [1,2]. BO is common, with prevalence reported to be as high as 6.8% amongst adults in the western world [3,4]. It is a premalignant condition, strongly associated with oesophageal adenocarcinoma (OAC) [2,5]. Patients have been reported to have an increased risk of OAC, that is as high as 60-fold compared to the normal population [2]. OAC has the fasting rising incidence of any solid organ cancer over the last 30 years in western countries and carries a particularly poor prognosis [6]. It is frequently diagnosed at a late stage with metastatic disease, and carries an overall five-year survival of 20%. For this reason, appropriate diagnosis and surveillance of BO is essential [1,3,4].

Surveillance guidelines published by the British Society of Gastroenterology (BSG) recommend surveillance endoscopy be performed between two to five yearly intervals, depending on the maximum length of BO [7]. Similar recommendations have also been made by the American Gastroenterology Association (AGA) [8,9]. Importantly, these guidelines apply to biopsy-proven diagnosis of BO rather than endoscopist suspicion based on appearance of the lower oesophagus during gastroscopy [7-9]. Biopsy protocols are well described. The Seattle protocol recommends stepwise four-quadrant biopsies of mucosa for every 2 cm of suspected BO [10,11].

BO surveillance is important, allowing for potential recognition and early treatment of high-grade dysplasia and intramucosal carcinoma [2,10,11]. However, gastroscopy is not a risk-free procedure, with a perforation rate of 0.06% [12]. As with any procedure, the benefits of performing the procedure must outweigh associated risks [12]. Thus, it is important that surveillance endoscopy is not performed without necessity. Despite the guidelines, many clinicians rely on endoscopic suspicion to give a diagnosis of BO before histology results are available, and it is not uncommon for BO surveillance endoscopy to be booked based on endoscopic findings rather than histological diagnosis [13]. This may lead to unnecessary investigation and inappropriate allocation of resources.

We set out to perform a retrospective review of all gastroscopy reports where an endoscopic diagnosis of BO was made over a one-year period in our centre. Follow-up recommendations at time of endoscopy were noted. Corresponding histology reports for patients were reviewed and compared to endoscopic findings. From this, we set out to establish the accuracy of upper gastrointestinal endoscopy in diagnosing BO prior to histological confirmation. This information may be useful in guiding practice towards scheduling surveillance based on histological diagnosis rather than endoscopic suspicion, thus allowing patients avoid unnecessary invasive procedures and to better allocate resources.

## Materials and methods

All upper gastrointestinal endoscopy reports in which a finding of BO was recorded were collected over a year long period in the endoscopy unit of a regional hospital. These were identified using the search functionality of EndoRaad^TM ^(MANITeX Limited, Dublin, Ireland), the electronic reporting system for endoscopy used in our centre, by using the search term ‘Barrett’s oesophagus’. All reports were collected and reviewed to confirm that diagnoses of BO were made by the endoscopist at the time of the procedure. Following this, reports were reviewed to assess whether or not lower oesophageal biopsies were taken at the time of the procedure. Patients who had a procedure in which lower oesophageal biopsies were not taken were excluded. Reports were also appraised for compliance with published guidelines for measuring and sampling suspected BO, such as the Prague criteria and the Seattle protocol [10,11,13]. The use of adjuncts such as narrow band imaging (NBI) and application of acetic acid chromoendoscopy at the time of procedure was also recorded. Other factors recorded from the endoscopy reports included speciality of the endoscopist (gastroenterology or general surgery), grade of the endoscopist (consultant or registrar), procedure length and quantity of sedation used to determine if these factors influenced results.

Histology reports were reviewed for all cases with endoscopic diagnosis of BO made with lower oesophageal biopsies taken at the time of the procedure. These reports were obtained using the hospital electronic histology database, with the patients’ medical record number used to search for the corresponding report. Final histology results were compared with endoscopy reports to determine the accuracy of an endoscopic diagnosis of BO. Only reports that were finalised by a consultant histopathologist were included as part of this study. Whether or not the speciality or grade of endoscopist, the length of time of the procedure, the quantity of sedation used, the use of NBI and documented use of published protocols impacted the accuracy of the diagnosis was also determined.

All patient data were anonymised for the purpose of this study. No identifying information was retained by the authors or included in this article. Statistical analysis was performed using the SPSS 24.0 software package (IBM Corp., Armonk, NY). A p-value of less than 0.05 was accepted as statistically significant.

## Results

Patient selection

During the study period, 102 patients underwent gastroscopy procedures in which a diagnosis of BO was recorded by the endoscopist. These procedures were performed between five gastroenterologists and six general surgeons. No lower oesophageal biopsies were taken in 18 procedures, which were subsequently excluded. This left 84 patients with an endoscopic diagnosis of BO and available histology results from lower oesophageal biopsies. Of these patients, 54 (64.2%) had their procedure performed by a gastroenterologist and 30 (35.8%) by a general surgeon. A total of 63 procedures (75%) were performed by consultants and 21 (25%) were performed by registrars. In terms of sedation, midazolam doses of 2 mg (n=9, 10.7%), 3 mg (n=14, 16.7%), 4 mg (n=38, 45.2%), 5 mg (n=9, 10.7%) and 6 mg (n=1, 1.2%) were recorded. No sedation was used in 13 patients (15.5%). The Seattle protocol was followed in 26 procedures (31%), with random lower oesophageal biopsies taken in the remaining 58 procedures (69%). The Prague classification system was used in 34 procedures (40.5%). The use of NBI/acetic acid chromoendoscopy was not recorded during any procedure (Table 1).

**Table 1:** Diagnostic accuracy of BO BO, Barrett’s oesophagus

Criteria	% incorrect diagnosis of BO	P value
Procedures performed by a gastroenterologist (n=54)	38.8% (n=21)	0.04093
Procedures performed by a general surgeon (n=30)	50% (n=15)	
Procedures performed by a consultant (n=63)	34.9% (n=22)	0.01078
Procedures performed by a registrar (n=21)	66.7% (n=14)	
Procedures with Prague classification (n=34)	29.4% (n=10)	0.04036
Procedures without Prague classification (n=50)	52% (n=26)	
Procedures with Seattle protocol (n=26)	26.9% (n=7)	0.0001
Procedures without Seattle protocol (n=58)	41.3% (n=24)	
Accuracy of BO diagnosis according to dose of midazolam used		
Dose of sedation	% correct diagnosis of BO	
No intravenous sedation	76.9% (n=10)	
2 mg intravenous midazolam	100% (n=9)	
3 mg intravenous midazolam	71.4% (n=10)	
4 mg intravenous midazolam	42.1% (n=16)	
5 mg intravenous midazolam	33.3% (n=3)	
6 mg intravenous midazolam	0% (n=0)	

Diagnostic accuracy of BO

Of the 84 patients who had a diagnosis of BO made at the time of the procedure, 48 patients (57.1%) had the diagnosis confirmed with the final histology result, while 36 (42.9%) were revealed to not have BO according to the specimens taken. Of the procedures with an incorrect diagnosis, 36.1% (n=13) were booked for surveillance endoscopy at the time of their procedure based on the assumptive diagnosis of BO. Of the procedures performed by a gastroenterologist with an endoscopic BO diagnosis, 38.8% (n=21) had histology negative for BO, compared to 50% (n=15) of those performed by a general surgeon. Of the procedures performed by a consultant with an endoscopic BO diagnosis, 34.9% (n=22) had a histology result that did not correlate with the endoscopic findings, compared to 66.7% (n=14) of procedures performed by a registrar. Diagnostic accuracy was higher during endoscopy where the Prague classification was used, with only 29.4% (n=10) of these being incorrect according to the histology. This is compared to 52% (n=26) amongst procedures where the Prague classification was not employed. Diagnostic accuracy at the time of gastroscopy was not improved with higher doses of intravenous sedation. The implementation of the Seattle protocol had a positive impact on diagnostic accuracy (Table 1).

## Discussion

The incidence of OAC has dramatically increased in the western world over the last two decades and is associated with a dismal prognosis [14]. Early diagnosis is essential to improve outcomes. BO is the only identified precursor lesion and most important risk factor for OAC [15]. Patients with BO have been shown to have a relative risk of OAC approximately 10-fold that of the general population [16]. While OAC is often a fatal diagnosis, treatment of BO with lifelong proton pump inhibitor (PPI) therapy can greatly reduce progression of BO to OAC, with a 75% reduction in the risk of neoplastic progression having been previously demonstrated [17]. Early recognition of low-grade and high-grade dysplasia can also allow for endoscopic interventions, such as endoscopic mucosal resection, radiofrequency ablation and photodynamic therapy, thus avoiding progression to adenocarcinoma [18]. For this reason, accurate diagnosis and surveillance of BO is essential.

However, our study clearly highlights that our ability to diagnose BO on endoscopic appearance alone is unreliable. The BSG defines BO as an endoscopically visible segment of columnar-lined oesophagus that has been histopathologically verified [7]. Despite this, it has been shown that it remains common practice to make the diagnosis at the time of endoscopy, and thus committing the patient to further surveillance procedures before the diagnosis is confirmed by the final histological report [13]. The EndoRaad system, which is in mainstream use for the purpose of writing endoscopy reports, prompts the endoscopist to make a diagnosis at the time of the procedure as well as a follow-up plan. This may make it tempting to the endoscopist to commit the patient to a further surveillance procedure at this time before histopathology is available, with possible failure to act on the later histology report and the potential medicolegal consequences of this possibly playing a role [13]. However, measures such as the implementation of a ‘virtual’ outpatient clinic, with a dedicated time period to review histology reports and arrange appropriate follow-up accordingly, can help alleviate this and ensure all final reports are acted upon [19].

However, such unnecessary procedures may have a significant detrimental impact on patient outcomes. Upper gastrointestinal endoscopy is not without risk, with both perforation and bleeding being recognised complications, particularly in the context of oesophageal biopsies [12]. An incorrect BO diagnosis may also commit a patient unnecessarily to lifelong PPI therapy, which is hard to justify in the context of increasing evidence of the numerous side effects of long-term PPI use [20]. Unnecessary procedures also put significant pressure on endoscopy waiting lists, resulting in unacceptably long waiting lists, potentially delaying serious diagnoses and having a detrimental effect on patient outcomes [21]. Both the Health Service Executive (HSE) and the National Health Service (NHS) have reported difficulty in achieving waiting time targets for urgent endoscopic procedures [21,22]. For these reasons, practices that lead to unnecessary procedures should be addressed.

Certain factors seem to play a role in the ability of an endoscopist to accurately predict a diagnosis of BO. Perhaps unsurprisingly, accuracy of endoscopic diagnosis was observed to be higher in more experienced clinicians, with consultants more accurately identifying BO than registrars. It was also observed to be higher according to the volume of procedures performed, with gastroenterologists more accurately predicting BO than surgeons. The use of the Prague classification improved accuracy of endoscopic diagnosis, perhaps being owed to the extra time spent observing the oesophagus to achieve accurate measurements. Interestingly, endoscopic diagnosis of BO appeared to be more accurate with lower doses of sedation. Implementation of the Seattle protocol also had a positive impact, with a greater volume of biopsies likely helping to avoid inadequate sampling. Nonetheless, while all these factors do show some impact on the accuracy of endoscopic diagnosis, the proportion of incorrect diagnoses was still high across all variables.

Our findings are important. Few studies have previously evaluated the ability to accurately endoscopically recognise BO. We have shown that, in a busy hospital with a high-volume endoscopy unit with multiple endoscopists, a presumptive diagnosis of BO was made based on endoscopic appearances in 102 cases over the course of a year. However, 42.9% of these patients did not have the diagnosis confirmed on their lower oesophageal biopsies, with a third of these patients booked at the time of their procedure for an unnecessary surveillance endoscopy. These inaccuracies appear to be high with both gastroenterologists and surgeons of all grades. This shows that our ability to accurately diagnose BO purely based on endoscopic views is unreliable. Despite this, it remains standard practice to do so [13].

## Conclusions

It is insufficient to rely on endoscopic appearances alone to diagnose BO, irrespective of speciality or experience. The diagnosis should only be made and surveillance endoscopy should only be scheduled after reviewing the histopathology report. The implementation of both the Prague classification and Seattle protocol can improve diagnostic accuracy. The implementation of virtual outpatient clinics can ensure these histology reports are appropriately acted upon and thus eliminate unnecessary repeat endoscopy procedures, sparing the patient from unjustifiable risk and cutting down on long waiting lists in endoscopy departments.

## References

[1] Spechler SJ, Souza RF (2014). Barrett’s esophagus. N Engl J Med.

[2] Williamson WA, Ellis FH, Gibb SP, Shahian DM, Thomas Aretz H, Heatley GJ, Watkins E Jr (1991). Barrett’s esophagus. Prevalence and incidence of adenocarcinoma. Arch Intern Med.

[3] Ronkainen J, Aro P, Storskrubb T (2005). Prevalence of barrett’s esophagus in the general population: an endoscopic study. Gastroenterology.

[4] Rex DK, Cummings OW, Shaw M (2003). Screening for Barrett’s esophagus in colonoscopy patients with and without heartburn. Gastroenterology.

[5] Brown LM, Devesa SS, Chow WH (2008). Incidence of adenocarcinoma of the esophagus among white Americans by sex, stage and age. J Natl Cancer Inst.

[6] Coleman HG, Xie S-H, Lagergren J (2018). The epidemiology of esophageal adenocarcinoma. Gastroenterology.

[7] Fitzgerald RC, di Pietro M, Ragunath K (2014). British Society of Gastroenterology guidelines on the diagnosis and management of Barrett’s oesophagus. Gut.

[8] Shaheen NJ, Richter JE (2009). Barrett’s oesophagus. Lancet.

[9] Shaheen NJ, Falk GW, Iyer PG, Gerson LB (2016). ACG clinical guidelines: diagnosis and management of Barrett’s esophagus. Am J Gastroenterol.

[10] Abela JE, Going JJ, Mackenzie JF, McKernan M, O'Mahoney S, Stuart RC (2008). Systematic four-quadrant biopsy detects Barrett’s dysplasia in more patients than nonsystematic biopsy. Am J Gastroenterol.

[11] Provenzale D, Schmitt C, Wong JB (1999). Barrett’s esophagus: a new look at surveillance based on emerging estimates of cancer risk. Am J Fastroenterol.

[12] Misra T, Lalor E, Fedorak RN (2004). Endoscopic perforation rates at a Canadian university teaching hospital. Can J Gastroenterol.

[13] Rehan JH, Magee C (2020). United European Gastroenterology. Mistakes in the endoscopic diagnosis and management of Barrett’s oesophagus and how to avoid them. United European Gastroenterology.

[14] di Pietro M, Peters CJ, Fitzgerald RC (2008). Clinical puzzle: Barrett’s oesophagus. Dis Model Mech.

[15] Wiseman EF, Ang YS (2011). Risk factors for neoplastic progression in Barrett’s esophagus. World J Gastroenterol.

[16] Hvid-Jensen F, Pedersen L, Drewes AM, Sorensen HT, Funch-Jensen P (2011). Incidence of adenocarcinoma among patients with Barrett’s esophagus. N Engl J Med.

[17] Kastelein F, Spaander MC, Steyerberg EW, Biermann K, Valkhoff VE, Kuipers EJ, Bruno MJ, ProBar Study Group (2013). Proton pump inhibitors reduce the risk of neoplastic progression in patients with Barrett’s esophagus. Clin Gastroenterol Hepatol.

[18] Eluri S, Shaheen NJ (2017). Barrett’s esophagus: diagnosis and management. Gastrointest Endosc.

[19] Healy P, McCrone L, Tully R (2019). Virtual outpatient clinic as an alternative to an actual clinic visit after surgical discharge: a randomised controlled trial. BMJ Qual Saf.

[20] Jaynes M, Kumar AB (2019). The risks of long-term use of proton pump inhibitors: a critical review. Ther Adv Drug Saf.

[21] (2020). The National Treatment Purchase Fund. IPDC GI Endoscopy by Group Hospital. https://www.ntpf.ie/home/inpatient_group.htm.

[22] Bowel Cancer UK (2020). Bowel Cancer UK. Unacceptable endoscopy waiting times put launch of new world-class screening programme at risk. https://www.bowelcanceruk.org.uk/news-and-blogs/news/unacceptable-endoscopy-waiting-times-put-launch-of-new-world-class-screening-programme-at-risk/.

